# Supplementary material for: Development and Field-Testing of Proposed Food-Based Dietary Guideline Messages and Images amongst Consumers in Tanzania
Source: Nutrients. 2022 Jun 29;14(13):2705. doi: 10.3390/nu14132705 (PMC9268523; doi:10.3390/nu14132705)
Supplement: Supplementary file 1 [file nutrients-14-02705-s001.zip › nutrients-1729709-supplementary.pdf]

FOCUS GROUP DISCUSSION SESSION OUTLINE

Welcome

Greet the participants and introduce yourself and the observers.
Thank the participants for their participation in this discussion.

Explanation of study

I will be asking you a number of questions relating to food-related guidelines. I will be doing this in order the gain information regarding how you feel about the guidelines and how well you understand them.
It shouldn't take too long (about 60-90 minutes). We will have a break in the middle of the session for refreshments.

Rules of engagement

- Please voice your opinions – whether positive or negative (there are no right or wrong answers)
- One person speaks at a time
- No interrupting – let the person finish before stating your own opinion
- There will be an audio-recorder during the session – please ignore it. I will not be able to remember everything that is said here today so I will use the recordings to analyse everything at a later stage. Remember, I will erase the recording once I have analysed the information.
- All of you have numbers placed on your shirts. This is how you will be identified – your names will not be used.

Informed consent

Before we can start, I need you all to provide informed consent. I will explain the procedure and then you will be required to sign the forms. Participation in this study is voluntary so it is up to whether or not you would like to participate in today's session. Bear in mind that you may withdraw at any time if you feel the need to do so.

Facilitator:
**hand out informed consent forms to participants*
**read through the form slowly*
**facilitate filling in of forms*
**if any participants are illiterate, assist them*
**collect all informed consent forms and check for completeness*

Socio-demographic questionnaire

I will need background information from all of you regarding your age, gender and socio-economic status. I will read through the questionnaire with you. Once I have read each question, please tick the appropriate box or write down your answer.

Facilitator:
**hand out questionnaire to participants*
**read through the questions slowly*
**facilitate answering of all questions*
**if any participants are illiterate, assist them*
**collect all questionnaires and check for completeness*

Ice-Breaker

Before we start, I would like everyone to introduce themselves and tell us something about yourself.

Discussion

We will now start with the discussion (*switch recorder on)

Observer/s:
Take note and write down the following information about the group during sessions:
**dynamics of the group*
**body language and facial expressions when hearing a guideline or the question*
**group agreement or disagreement regarding a question/answer*

FOR EACH GUIDELINE, ASK THE FOLLOWING QUESTIONS

- Have you heard about or read this guideline before?
    - If yes, where?

- What do you understand by this guideline?
    - Prompt: What is this guideline trying to tell you?
    - Prompt: What is this guideline trying to explain to you?
    - Prompt: What does this guideline mean to you?

- Do you think the guideline is important?
    - If Yes – why?
    - If No – why not?

- Do you think you will be able to follow this guideline?
  - If Yes – why?
  - If No – why not?

- Do you think the general public will be able to understand this guideline?
  - If Yes – why?
  - If No – why not?

- How would you re-word this guideline to make it more understandable to the general public?

FOR EACH FOOD GUIDE IMAGE, ASK THE FOLLOWING QUESTIONS

- What do you understand by this image?
  - Prompt: What is this picture trying to tell you?
  - Prompt: What is this picture trying to explain to you?
  - Prompt: What does this picture mean to you?

- Do you think the image/picture and the guideline complement each other?
  - If Yes – why?
  - If No – why not?

- Do you think the general public will be able to understand this image?
  - If Yes – why?
  - If No – why not?

- How would you change this image/picture to make it more understandable to the general public?

Closing

I would like to thank you all again for participating in today's session.

SELF-ADMINISTERED QUESTIONNAIRE FOR PARTICIPANTS

Code: ______

Date (dd/mm/yyyy): ___________

District:
- ☐ Dar es Salaam
- ☐ Mwanza
- ☐ Iringa
- ☐ Kusini (Zanzibar)

INSTRUCTIONS:

a) Please answer all of the questions.

b) Please complete the questionnaire below by ticking the appropriate box, circling the most appropriate number or writing the answer in the space provided.

DEMOGRAPHIC INFORMATION:

1) Date of Birth: (day/month/year): …………………..

2) Home language:
- ☐ Kiswhahili
- ☐ Other: …………………………………..

3) Highest level of education:
- ☐ None
- ☐ Grade 1 – Grade 7
- ☐ Grade 8 – Grade 11
- ☐ Grade 12
- ☐ Diploma
- ☐ Tertiary education (technicon, university)

4) Employment status:
- ☐ Employed
- ☐ Unemployed looking for work
- ☐ Unemployed, not looking for work

5) Do you have children: ☐ Yes ☐ No

If Yes:
- Number of children: ☐

- Ages of children: ………………………………………
………………………………………………….

6) What is your role relating to food in the household:
You can tick more than one box. Please tick all the applicable boxes:

☐ Provide or contribute money for food
☐ Decides what food should be bought or used in the house
☐ Purchase food
☐ Prepares food
☐ Grow food for the use in the household
☐ Grow food and sell produce for money
☐ Other (Please specify
…………………………………………………………………
…………………………………………………………………
